# Genetics of Schizophrenia: Overview of Methods, Findings and Limitations

**DOI:** 10.3389/fnhum.2017.00322

**Published:** 2017-06-22

**Authors:** Mads G. Henriksen, Julie Nordgaard, Lennart B. Jansson

**Affiliations:** ^1^Mental Health Center Glostrup, University Hospital of CopenhagenCopenhagen, Denmark; ^2^Faculty of Health and Medical Sciences, Institute of Clinical Medicine, University of CopenhagenCopenhagen, Denmark; ^3^Center for Subjectivity Research, University of CopenhagenCopenhagen, Denmark; ^4^Early Psychosis Intervention Center, Region Zealand Psychiatry Roskilde, University of CopenhagenCopenhagen, Denmark

**Keywords:** twin, adoption, linkage, candidate-gene, GWAS, CNVs, SNVs, self-disorders

## Abstract

Genetics constitute a crucial risk factor to schizophrenia. In the last decade, molecular genetic research has produced novel findings, infusing optimism about discovering the biological roots of schizophrenia. However, the complexity of the object of inquiry makes it almost impossible for non-specialists in genetics (e.g., many clinicians and researchers) to get a proper understanding and appreciation of the genetic findings and their limitations. This study aims at facilitating such an understanding by providing a brief overview of some of the central methods and findings in schizophrenia genetics, from its historical origins to its current status, and also by addressing some limitations and challenges that confront this field of research. In short, the genetic architecture of schizophrenia has proven to be highly complex, heterogeneous and polygenic. The disease risk is constituted by numerous common genetic variants of only very small individual effect and by rare, highly penetrant genetic variants of larger effects. In spite of recent advances in molecular genetics, our knowledge of the etiopathogenesis of schizophrenia and the genotype-environment interactions remain limited.

## Introduction

Despite a century of research, our knowledge of the etiology and pathogenetic unfolding of schizophrenia remains scarce. A persistent scientific problem may have several overlapping sources: it may be due to the intrinsic difficulty of the object of inquiry, to methodological or technological inadequacies, or to a mistaken formulation of the research problem. As we shall see, some of these sources have played a role in the history of research on schizophrenia genetics.

In the last decade, genetic research in schizophrenia has experienced a new dawn infused by a regained optimism due to newly developed, far more advanced molecular, technological and statistical methods. Given the rapid progress and intrinsic complexity of molecular genetic research (reflected, e.g., in the technical language of many molecular genetic studies), it may be difficult for outsiders to the field to grasp and appreciate the results from studies on schizophrenia genetics. Since genes are considered the strongest risk factor for schizophrenia, some grasp of this complex research domain is relevant in many clinical contexts.

The purpose of this article is to contribute to facilitate such an understanding by providing an accessible overview of some of the central methods and findings in genetic research in schizophrenia, from its historical origins to current status. In other words, we are not offering a comprehensive review of the entire field but a brief overview that may provide the reader with an initial orientation in the field. For this reason, we generally refrain from discussing the details of the manifold findings in especially molecular genetics. Finally, we seek to articulate certain limitations and challenges that tend to be deemphasized in this field of psychiatric research.

## Models of Genetic Transmission

It has for a long time been known that madness (and many other human afflictions and characteristics) runs in families. After Mendel’s discovery of the laws of monogenic transmission of phenotypic traits, some of the earliest authors, describing schizophrenia, assumed an inherited basis of schizophrenia risk due to familial aggregation of the disease or its milder variants (Bleuler and Jung, 1908). The monogenic model of schizophrenia was attractive for a variety of reasons, e.g., simplicity, a hope of discovering a corresponding, simple pathophysiological mechanism, and because it fitted into available theoretical options (i.e., recessive, dominant, with varying penetrance). The strictly monogenic theory was, however, quickly abandoned, because it did not fit the empirical data (even with the quantitative help of the concept of penetrance). Yet, the very idea of one specific gene or, later, a few specific genes as being etiologically necessary but not sufficient for the emergence of schizophrenia survived until fairly recently. For example, Meehl (1962) believed in a monogenic necessary gene, whose action was modified by polygenic factors. Holzman (1989) proposed the “latent trait model”, suggesting that a dominant gene results in a latent trait, a postulated neural deficit with potentially pleiotropic manifestations (e.g., schizophrenia, schizotypy or eye-movement disorder). Risch and Baron (1984) offered the “mixed model”, claiming that a specific gene in combination with a few oligogenes and a polygenic-multifactorial background formed the genetic substrate. All these models have been tried to fit, with varying degree of success, to the available epidemiological data of schizophrenia. In this context, it merits special attention that Gottesman and Shields (1967) already proposed a polygenic model for schizophrenia. As we shall see, research in molecular genetics documents that schizophrenia is in fact best accounted for by complex, polygenic model.

## Pre-Molecular Genetics

In the first half of the 20th century, family studies demonstrated that the rate of schizophrenia was higher in relatives of patients with schizophrenia than in the general population (Rüdin, 1916; Kahn, 1923; Schulz, 1932; Kallmann, 1938). Twin studies documented that the concordance rate (i.e., both twins suffering from schizophrenia) was elevated in monozygotic (MZ) twins compared to dizygotic (DZ) twins (Luxenburger, 1928; Kallmann, 1946; Slater, 1953). These early twin studies were later criticized for various methodological reasons (Rosenthal, 1959, 1962; vide infra). From the 1960s, improved twin (Kringlen, 1967; Fischer, 1973) and adoption studies (Heston, 1966; Rosenthal et al., 1968; Kety et al., 1975; Tienari et al., 1985) became crucial in determining the familial clustering and concordance rates for schizophrenia. By indicating a strong genetic component in the etiology of the illness, the studies contributed to undermine the psychoanalytical hypothesis of schizophrenic causation, claiming that schizophrenogenic rearing was either a necessary or sufficient cause for developing schizophrenia. The basic intuition behind the twin studies is the following: given that MZ twins (sharing 100% of their genes) and DZ twins (sharing 50% of their genes) share the environment they are raised in, higher concordance rates in MZ over DZ twins most likely result from genetic similarity. Estimates of concordance rates for schizophrenia, based on European twin studies from 1963 to 1987, show higher rates for MZ (48%) than for DZ twins (17%; Gottesman, 1991), and similar concordance rates were reported in European and Japanese twin studies from 1992 to 1999—41%–65% for MZ vs. 0%–28% for DZ twins (Cardno and Gottesman, 2000). A meta-analysis (Sullivan et al., 2003) of twin studies estimates the genetic liability to schizophrenia at 81% (95% CI, 73%–90%), whereas shared environmental influences were estimated to be 11% (95% CI, 3%–19%). Finally, a few studies of children of discordant MZ twins found a similar risk of schizophrenia spectrum disorders in the children of the affected and unaffected MZ twin (Gottesman and Bertelsen, 1989; Kringlen and Cramer, 1989), presumably indicating that unaffected MZ twins carry silent (non-expressed) susceptibility genes for schizophrenia. By contrast, for children of discordant DZ twins, the risk was higher in the children of the affected DZ twin compared to the children of the unaffected DZ twin (Gottesman and Bertelsen, 1989).

Adoption studies have documented that schizophrenia spectrum disorders are more frequent in adopted-away children of mothers with schizophrenia than in their control adoptees (Heston, 1966; Rosenthal et al., 1968; Kety et al., 1975, 1994). A cross-fostering study (Wender et al., 1974) found that children of healthy parents, adopted by a family where one of the parents later developed schizophrenia, did not have an increased risk of developing schizophrenia. Other studies (Heston, 1966; Higgins, 1976) found that children of mothers with schizophrenia had the same risk of developing the disorder independent of whether they were raised by their biological mothers or by adopting parents with no history of mental illness. A Finish adoption study (Tienari et al., 1985, 2004) found that markedly dysfunctional rearing environments (the adoptive families were initially assessed and classified on a scale ranging from “1. healthy” to “5. severely disturbed”) predicted schizophrenia spectrum disorders in adopted-away children of mothers with schizophrenia but not in their genetically undisposed controls. Interestingly, similar results were reported in the Danish High-Risk study (Mednick et al., 1987), which found increased risk of schizophrenia in children of mothers with schizophrenia, who were exposed to unstable parenting or raised in public childcare institutions (Parnas et al., 1985).

## Molecular Genetics

The Human Genome Project (1990–2003) has been instrumental in molecular genetic research in schizophrenia. The Human Genome Project was an international research effort to determine the sequence of the human genome’s three billion base pairs and to map all of its genes. At the dawn of molecular genetics in the early 1980s, some researchers, though certainly not all, believed that within a fairly limited period of time the availability of DNA would reveal the biological causes of the disorder (e.g., Andreasen, 1984), as jointly indicated by twin and adoption studies.

The first DNA-based method was “linkage analysis”, which aimed at discovering genomic regions in samples of affected extended or nuclear families and sibling pairs without implicating a specific allelic variant. By examining the degree of co-segregation of genetic markers and predefined phenotypic traits (e.g., schizophrenia spectrum diagnosis), estimates of linkage between the illness and genomic loci were obtained. Linkage analysis is based on the observation that genetic markers, which are located physically close on the same chromosome, tend to be inherited together, i.e., they remain “linked” during meiosis. Numerous linkage studies of schizophrenia have been conducted, but positive findings have generally proved difficult to replicate in subsequent studies (Risch and Merikangas, 1996). In brief, results from meta-analyses (Badner and Gershon, 2002; Lewis et al., 2003; Ng et al., 2009) suggest that many chromosomal regions may contain schizophrenia susceptibility loci. Notably, these loci do not themselves confer risk but they may harbor variants that do. These results also made it clear that the power of the linkage design was too weak to address genomic loci with small effects; the sample size requirement necessary to detect linkage was simply practically unachievable (Risch and Merikangas, 1996). Hence, other DNA-based methods were required to key in on the genes potentially involved in the etiology of schizophrenia.

The next wave of molecular genetic research in schizophrenia employed the “candidate gene” approach, which, using a case-control study design, explored if potential susceptibility genes correlate with the disorder. In contrast to linkage analysis, the candidate gene approach can detect genes with small effect alleles provided that the sample size is adequate. Candidate genes have usually been selected due to their position (e.g., from findings in linkage analyses) or functionality (e.g., genes coding for proteins related to dopamine or serotonin neurotransmission). Today, more than 1000 candidate genes have been tested (for details see http://www.szgene.org) but despite identification of some genes with small effect alleles (see e.g., Haraldsson et al., 2011), the overall results from the candidate gene studies have been disappointing (Gejman et al., 2011). Some of the most cited candidate genes are *DISC1*, *DTNBP1*, *NRG1* and *COMT*, but their potential pathogenetic involvement in schizophrenia remains debated. The absence of significant discoveries may have several reasons, e.g., difficulties in replicating positive findings, inadequate statistical power, and limited knowledge of the genes believed to be involved in the pathophysiology of schizophrenia (which obviously makes it difficult to select relevant candidate genes for testing).

In contrast to the hypothesis-driven candidate gene approach that typically could test only relatively few genetic markers in delimited genomic loci in each study, the genome-wide association studies (GWAS), which also often employ a case-control study design, interrogate the genome purely empirically (i.e., GWAS do not rely on any* a priori* selected candidate genes) for associations between common genomic variants or loci and the disorder. The identification and mapping of millions of common single nucleotide polymorphisms (SNPs), as facilitated by initiatives such as the International HapMap Project and the 1000 Genomes Project (continued by The International Genome Sample Resource), has been instrumental for the GWAS approach. GWAS are based on linkage disequilibrium, i.e., a non-random association of alleles at two or more loci. Recent technological advances such as microarrays and chips have made it possible to quickly and inexpensively scan a million SNPs genome-wide. The reasoning behind the GWAS approach is that if specific allele variants are found more frequently in patients than in their controls, then the allele variants may be indicative of a genetic association. To minimize the risk of Type I errors (i.e., false positives), most GWAS operate with a stringent threshold of significance (*p* < 5 × 10^−8^). Since 2007, schizophrenia GWAS have been published (for details see http://www.genome.gov/gwastudies). Overall, the studies have failed to support the findings from linkage and candidate gene studies, but the GWAS have instead identified a large number of new susceptibility loci of only very small individual effects—and many of these genomic loci have in fact been replicated in subsequent GWAS and have reached meta-analytic genome-wide significance (see e.g., Shi et al., 2009; Stefansson et al., 2009; Schizophrenia Psychiatric Genome-Wide Association Study Consortium, 2011; Aberg et al., 2013; Ripke et al., 2013; Xiao and Li, 2016; Yu et al., 2016). One seminal study (Schizophrenia Working Group of the Psychiatric Genomics Consortium, 2014) combined available schizophrenia GWAS samples into a single analysis and successfully identified 128 independent schizophrenia associations, spanning 108 risk loci of genome-wide significance, 83 of which were novel findings. For example, associations were found at dopamine receptor D2, in several genes involved in glutamatergic neurotransmission and synaptic plasticity, and in tissues with central immune functions. The authors suggest that these results provide some genetic support for the hypothesized links between schizophrenia and dopamine and immune dysregulation, respectively.

Furthermore, associations have repeatedly been found between schizophrenia and genetic markers across the extended Major Histocompatibility Complex (MHC) locus on chromosome 6 (25–34 Mb), implicating the MHC locus as strongest of the >100 loci of genome-wide significance (see e.g., Shi et al., 2009; Stefansson et al., 2009; Schizophrenia Psychiatric Genome-Wide Association Study Consortium, 2011; Schizophrenia Working Group of the Psychiatric Genomics Consortium, 2014). The MHC locus is known to harbor genes with immune functions and attempts to link the locus to schizophrenia date back to the 1970s (Gejman et al., 2011). A recent study (Sekar et al., 2016) found that the association between schizophrenia and the MHC locus to a considerable extent stems from many common, structurally distinct alleles of the complement component 4 (*C4*), and these alleles were moreover found to affect the expression of *C4A* and *C4B* in the brain and to be associated with schizophrenia in proportion to their effect on *C4A* expression. Finally, it merits attention that several GWAS have found shared genetic risk loci in schizophrenia and bipolar disorder (e.g., Moskvina et al., 2009; Schizophrenia Psychiatric Genome-Wide Association Study Consortium, 2011; Cross-Disorder Group of the Psychiatric Genomics Consortium, 2013; Sleiman et al., 2013); we discuss these findings in the section on limitations and challenges.

The rationale behind GWAS is the “common-disease common-variants” hypothesis, which suggests that schizophrenia is mainly associated with common genetic variants (SNPs). As we have seen, large-scale GWAS have identified more than 100 risk loci.

However, it merits attention that a seminal study (International Schizophrenia Consortium et al., 2009) demonstrated that a substantial polygenic component of schizophrenia risk is in fact not to be found in a large number of strongly associated loci but rather in thousands of common alleles of only a very small effect that individually do not attain significance. The predictive accuracy of polygenic risk scores is likely to further improve as sample sizes continue to grow (Dudbridge, 2013). Still, there is an increasing awareness that common variants only explain a proportion of the heritability of schizophrenia, which refers to the proportion of variance between individuals that is accounted for by genetic factors. Individually, most of these common alleles confer only relatively small risk (typically odds ratios <1.2) but cumulatively they have been estimated to explain between a quarter and half of the variance in genetic liability (e.g., International Schizophrenia Consortium et al., 2009; Lee et al., 2012; Ripke et al., 2013; Arnedo et al., 2015). In other words, a proportion of the variance in genetic liability is apparently not accounted for by common genetic variants. Addressing this issues, the “common-disease rare-variants” hypothesis (McClellan et al., 2007) proposes that highly penetrant, rare (<1%) genetic variants, including copy number variations (CNVs), single nucleotide variants (SNVs), and small insertions and deletions (indels), contribute to the genetic component of schizophrenia. The two hypotheses are complementary to each other. In the following, we briefly address some of the most significant rare genetic variants, which, in the last few years, substantially have increased our understanding of the spectrum of genetic risk variants.

First, there is now strong evidence that rare, *de novo* (i.e., new, not inherited) or inherited CNVs, i.e., structural genomic variants that consist primarily of duplication or deletion, confer high risk for schizophrenia. CNVs range in size from one kilobase (kb) to several megabase (Mb) pairs. Several studies have found elevated levels of rare CNVs in patients with schizophrenia compared to controls (International Schizophrenia Consortium, 2008; Xu et al., 2008; International Schizophrenia Consortium et al., 2009; Malhotra et al., 2011; Szatkiewicz et al., 2014; Chang et al., 2016; Ruderfer et al., 2016). For example, robust associations have been uncovered between schizophrenia and rare, large (>100 kb) CNVs, including deletions on chromosome 1q21.1, 3q29, 15q13.3 and 22q11.2, and duplications on chromosome 16p11.2 and 16p13.11—the odds ratios of these CNVs range from approximately 2 to 60 (Rees et al., 2015). Moreover, deletions of *NRXN1* have been substantially linked to schizophrenia (e.g., Kirov et al., 2009).

Second, exome sequencing, a technology that allows for identification of DNA variants within the 1% protein-coding regions or genes (exons) of the genome (the exome), has enabled scans of genes for mutations at single-base resolution, which previously could not be detected, i.e., SNVs and indels. The rationale behind exome sequencing is that variations in these sequences are likely to entail more severe consequences than variations in the remaining 99% of the genome. Several studies have now used exome sequencing to explore SNVs and indels in schizophrenia. Some studies have reported a slightly increased exome-wide level of rare and/or *de novo* SNVs in patients with schizophrenia compared to controls (Xu et al., 2012; McCarthy et al., 2014a, 2016) but this finding has not been replicated in larger studies (Fromer et al., 2014; Purcell et al., 2014). Interestingly, Fromer et al. (2014) found *de novo* SNVs and indels to be significantly enriched in glutamatergic postsynaptic proteins, comprising the ARC (activity-regulated cytoskeleton-associated protein) and N-methyl-D-aspartate receptor (NMDAR) postsynaptic protein complexes, which previously have been linked to schizophrenia in CNV studies (Glessner et al., 2010). Finally, Purcell et al. (2014) used exome sequencing to explore rare SNVs and indels in schizophrenia and found a polygenic burden of very rare (<1/10,000), disruptive variants distributed across many genes in a set of 2546 genes previously implicated in schizophrenia by GWAS, and CNV and *de novo* SNV studies (see Richards et al., 2016).

In sum, pre-molecular and molecular genetics have demonstrated beyond doubt that genetics constitute a strong risk factor for schizophrenia. In contrast to the initial monogenic and oligogenic models of genetic transmission, there is now compelling evidence that the genetic architecture of schizophrenia is very complex, heterogeneous, and polygenic—the disease risk is constituted by numerous common genetic variants of only very small individual effects (e.g., SNPs) and by uncommon, highly penetrant genetic variants of larger effect (e.g., CNVs).

## Limitations and Challenges

As any research question, pre-molecular and molecular genetic studies in schizophrenia are based on certain assumptions and confront various limitations and challenges that must be made explicit if we are to properly appreciate the empirical findings. In the following, we discuss what we believe are six of the most important ones.

First, the classical twin design remains controversial and its validity has regularly been called into question (e.g., Charney, 2012; Turkheimer and Harden, 2014). Although the intuition behind the twin studies seems straightforward (*vide supra*), it is, in fact, not unproblematic. In order to take the higher concordance rates in MZ than in DZ twins as evidence for a genetic component, some fairly unlikely assumptions are required, e.g., we must statistically hold the environment constant, i.e., we must assume that the environments experienced by MZ and DZ twins do not differ in any way that may be relevant for the development of schizophrenia; and we must assume that genes and environment are both mutually independent and jointly additive (inclusive) for the development of schizophrenia. The problem with the classical twin design is that many, if not most, behavioral traits seem to act quite similarly, i.e., definitely heritable with some variance ascribable to the non-shared environment and little to the shared environment. Notably, these remarks do not undermine the identified concordance rates for schizophrenia in MZ and DZ twins, but they do put into perspective the problem of making inferences and estimations of the size of the genetic component in schizophrenia on the basis of the classical twin design. Although the classical twin design does not play a major role in genetic studies today, estimates of the genetic contribution to schizophrenia, based on previous twin studies, are often stated as facts in many textbooks and research articles on schizophrenia, and therefore we believe it is still important to voice these concerns.

Second, a challenge confronting molecular genetic research is, in our view, the apparent variability in the clinical manifestation of schizophrenia and the absence of a biomarker to compensate for the shortcomings in phenotypic demarcation. According to Baron (2001), attempts to circumvent this problem have involved dissecting schizophrenia into clinical subtypes aggregating in families (e.g., periodic catatonia), replacing the phenotype (schizophrenia) with symptom-based analysis (e.g., positive and negative symptoms) or endophenotypes (e.g., impaired sensory gating and ocular movement dysfunction), and blurring the diagnostic boundaries between schizophrenia and other major mental disorders (e.g., bipolar disorder). The elimination of diagnostic boundaries has led to potentially interesting genetic findings indicative of an overlap of genetic susceptibility loci between schizophrenia and bipolar disorder (Moskvina et al., 2009; Schizophrenia Psychiatric Genome-Wide Association Study Consortium, 2011; Cross-Disorder Group of the Psychiatric Genomics Consortium, 2013; Sleiman et al., 2013). These results are somewhat surprising given that family studies usually have found that these disorders do not co-aggregate in families (Kendler et al., 1993; Maier et al., 1993). Yet, a large, population-based study of approximately 75,000 affected Swedish families with schizophrenia or bipolar disorder found a co-aggregation in the families, providing some epidemiological support for the hypothesis of an at least partially shared genetic basis (Lichtenstein et al., 2009). Crucially, however, this study was based on hospital discharge rather than research diagnoses, and we may speculate if the apparent co-aggregation perhaps could result from different diagnostic practices.

Third, it merits attention that the symptom-based analysis, the blurring of diagnostic boundaries, the case-control design of many GWAS, CNV and exome sequencing studies, and the detection of shared genetic risk loci between schizophrenia, bipolar disorder, and sometimes also autism is indicative of a genetic vulnerability to mental disorders more broadly and not to schizophrenia specifically (i.e., genetic pleiotropy). While identifying shared genetic vulnerability is crucial in its own right, keying in on what is specific for schizophrenia presents an obvious target for contemporary and future molecular genetic research. One way of keying in on what is specific to schizophrenia is illustrated in a GWAS (Ruderfer et al., 2014), where the authors explored the discriminability of schizophrenia from bipolar disorder and found that no SNPs reached genome-wide significance but, on the basis of computed risk scores, the authors identified a polygenic signal capable of discriminating schizophrenia from bipolar disorder. In this context, it also merits attention that a study of relatives of high-density schizophrenia families in Ireland found molecular support for the concept of the schizophrenia spectrum and its genetic basis (Bigdeli et al., 2014).

Fourth, another challenge concerns the implications of the molecular genetic findings, i.e., how do we obtain scientific knowledge of the effects of the, e.g., now >100 susceptibility loci that have reached genome-wide significance and their possible involvement in the etiology of schizophrenia? Is an empirical, bottom-up approach, systematically eliciting the biological functions related to each risk locus at all a negotiable road in this case? The prospect of studying all identified loci, singly and in potential mutual interactions, could turn into an infinite task. Moreover, if common genetic variation implicates an intractable amount of genes of only very small individual effect alleles, we may find ourselves in a situation, where, as Goldstein (2009) put it, “in pointing at everything, genetics would point at nothing”. Here, it seems that psychiatry may need assistance from systems biology to convert a multitude of genes of small effect alleles into a graspable and identifiable pathogenetic stream or field of study (Sauer et al., 2007; McCarthy et al., 2014b).

Fifth, some authors have used the apparent overlap of genetic susceptibility loci between schizophrenia and bipolar disorder as a lever to criticize the clinical validity of the Kraepelinian dichotomy (e.g., Owen et al., 2007; Lichtenstein et al., 2009; Doherty and Owen, 2014). The perpetual rebirth of the unitary view of psychosis is perhaps its clearest manifestation. Another expression of the dissatisfaction with the current psychiatric classification and the lack of etiological progress is found in the Research Domain Criteria (RDoC), which ultimately seeks to found psychiatric nosology on advances in genetics, neuroscience, behavioral sciences, etc., i.e., by disregarding the diagnostic categories of DSM-5 (American Psychiatric Association, 2013) and ICD-10 (World Health Organization, 1992). More generally, this criticism raises a crucial question, viz. what defines a mental disorder? Should we begin to understand psychosis on the basis of specific genetic profiles or on the basis of clinical phenotypes? Opting for a genetically (and biologically) informed remodeling of psychiatric nosology (e.g., as described by Insel and Cuthbert, 2015), founded upon i.a. our limited knowledge of certain susceptibility loci’s potential involvement in the etiology of various mental disorders, appears self-defeating for a number of diagnostic, therapeutic and epistemological reasons. In our view, no diagnostic classification in psychiatry can remain indifferent to the relevant clinical phenotypes, i.e., the patients’ suffering, experience and existence.

The final issue that we raise here is nosological and psychopathological in nature and it offers another perspective on how to key in on what is specific for schizophrenia, which also has relevance for genetic research. In this context, it merits attention that there are many schizophrenia definitions (Jansson and Parnas, 2007; Kendler, 2016) and most of these describe a relatively unspecific psychotic “end product” far away from the fundamental neurophysiological disturbances that assumingly are closer to the genetic basis of the disorder. In other words, psychiatric nosology carves phenotypes that have implications for research, and it is possible that the reification of the schizophrenia phenotype, which occurred with the so-called “operational revolution” in psychiatry in DSM-III (American Psychiatric Association, 1980), has in fact impeded rather than fostered research progress in schizophrenia (Parnas and Jansson, 2015). For example, the current schizophrenia concept in DSM-5 and ICD-10 defines the disorder as a primarily delusional-hallucinatory clinical phenotype—a definition that is remarkably different from Bleuler’s original concept of schizophrenia. Bleuler (1950) famously distinguished between “fundamental” and “accessory” symptoms, arguing the former are essential to schizophrenia, whereas the latter are not. On his account, delusions and hallucinations were considered as accessory symptoms—these symptoms are typically episodic in nature, they can be entirely absent, and they may also be found in other disorders. By contrast, the fundamental symptoms exhibit a trait-like quality—“[they] are present in every case and at every period of the illness” (Bleuler, 1950, p. 13). The fundamental symptoms include disturbances of association (formal thought disorders), ambivalence, autism and experiential ego-disorders, etc. Keenly aware of the poly-symptomatology of schizophrenia, Bleuler argued that the decisive diagnostic factor, separating schizophrenia from manic or depressive psychosis, is the presence of fundamental symptoms (Bleuler, 1950, p. 304). With the exception of severe forms of formal thought disorders, Bleuler’s fundamental symptoms and thus the core, trait-phenotypic features of schizophrenia were ignored in DSM-III and subsequent editions of the DSM as well as in ICD-10.

The theoretical and empirical research on anomalous self-experiences (“self-disorders”) can to some extent be seen as a return to and a systematic succession of a Bleulerian approach to psychopathology, i.e., the research focus is once more directed towards certain specific, non-psychotic, trait-like features of schizophrenia. However, where Bleuler’s (1950) fundamental symptoms largely were expressive features (signs), observable by the clinician, research on self-disorders elicits certain subjectively lived experiential anomalies (symptoms). For clinical descriptions of self-disorders in schizophrenia spectrum disorders, see Parnas and Handest (2003), Parnas et al. (2005a), Henriksen and Parnas (2012), and Henriksen and Nordgaard (2016). During the last two decades, empirical research on self-disorders consistently demonstrate: (i) that self-disorders hyper-aggregate in schizophrenia spectrum disorders but not in other mental disorders, including bipolar disorder (Parnas et al., 2003; Parnas et al., 2005b; Raballo et al., 2011; Haug et al., 2012; Raballo and Parnas, 2012; Nordgaard and Parnas, 2014), (ii) that self-disorders occur in genetically high-risk individuals (Raballo and Parnas, 2011), (iii) that self-disorders are temporarily stable over a 5-year period (Nordgaard et al., 2017); and finally (iv) prospective studies indicate that self-disorders predict transition to psychosis in an Ultra-High Risk for psychosis sample (Nelson et al., 2012) and that high baseline scores of self-disorders predict later transition to a schizophrenia spectrum diagnosis (Parnas et al., 2011, 2016)—for a review see Parnas and Henriksen (2014). Recently, self-disorders have been empirically explored as an intermediate phenotype of schizophrenia. Especially, discovering the neurophysiological correlates of self-disorders is already a topic of intense research. Several studies now point to a disturbance of emotional motor resonance and multisensory integration impairment as body-level correlates of self-disorders (e.g., Sestito et al., 2013, 2015a,b, 2017; Ebisch and Gallese, 2015). These studies show the potential of applying self-disorders as a target phenotype for neurobiological and also genetic research in schizophrenia.

## Conclusion

Pre-molecular and molecular genetic studies have demonstrated that genetics form a strong risk factor for schizophrenia. Many findings from schizophrenia GWAS have been replicated and several of these findings have reached meta-analytic genome-wide significance. The robust associations between schizophrenia and the >100 susceptibility loci, the identified CNVs and SNVs, respectively, seem promising on a number of scores. Also, the importance of the thousands of common alleles of only a very small effect, which do not individually achieve significance but which collectively form a substantial polygenic component of schizophrenia risk, should not be underestimated. Hopefully, these results will pave the way to truly novel, actionable, therapeutic knowledge. However, we should not fail to also notice: (i) that associations between common (SNPs) or uncommon (CNVs, SNVs) genetic variants and schizophrenia, though statistical facts, are not necessarily indexes of causal pathways; and (ii) that many of the discovered associations are, in fact, non-specific to schizophrenia but indicative of a genetic vulnerability to several mental disorders. Overall, the details of the etiopathogenesis of schizophrenia and the genotype-environment interactions remain to large extent unknown, and therefore caution is still warranted when drawing conclusions about the size of the genetic contribution in the etiology of the disorder.

## Author Contributions

MGH, JN and LBJ planned the study collectively. All authors contributed to the design, analyses and discussion. MGH wrote the first draft and all authors participated in critical revisions of the draft. All authors approved the final version and made agreement to be accountable for all aspects of the work in ensuring that questions related to the accuracy or integrity of any part of the work are appropriately investigated and resolved.

## Conflict of Interest Statement

The authors declare that the research was conducted in the absence of any commercial or financial relationships that could be construed as a potential conflict of interest.
